# Editorial: Importance of body composition analysis in clinical nutrition

**DOI:** 10.3389/fnut.2022.1080636

**Published:** 2023-01-12

**Authors:** Alberto Bazzocchi, Silvia Gazzotti, Lidia Santarpia, Clelia Madeddu, Maria Letizia Petroni, Maria Pilar Aparisi Gómez

**Affiliations:** ^1^Diagnostic and Interventional Radiology, IRCCS Istituto Ortopedico Rizzoli, Bologna, Italy; ^2^Department of Clinical Medicine and Surgery, Federico II University School of Naples, Naples, Italy; ^3^Department of Medical Sciences and Public Health, University of Cagliari, Cagliari, Italy; ^4^IRCCS-S. Orsola-Malpighi Hospital, Department of Medical and Surgical Sciences, University of Bologna, Bologna, Italy; ^5^Department of Radiology, Auckland City Hospital, Auckland, New Zealand; ^6^Department of Radiology, IMSKE, Valencia, Spain

**Keywords:** body composition analysis, clinical nutrition, metabolism, imaging, prognosis, therapeutic management

Body composition analysis (BCA) refers to the description and quantification of the various components that make up the human body. Body composition (BC) can be studied at five different levels: atomic, molecular, cellular, organ and tissue, and whole body level (1). In clinical nutrition, it is critical to distinguish fat mass (FM) from fat-free mass (FFM), including skeletal muscle (SM) mass. In addition, it is important to consider the distribution of fat mass (2), acknowledging that visceral adipose tissue (VAT) and subcutaneous adipose tissue (SAT) show relevant variations in structure and endocrine function and thereby have a different impact on cardiometabolic and cancer risk (3, 4). Of relevance, alterations in specific body components, such as depletion of skeletal muscle mass and loss of bone mineral density, may impact patient function and performance, as well as prognosis. BCA can be accomplished using a variety of techniques, such as anthropometric measurements, bioelectrical impedance analysis (BIA), dual-energy X-ray absorptiometry (DXA), computed tomography (CT), magnetic resonance imaging (MRI), and ultrasound imaging (US). Anthropometric measurements, including body mass index (BMI), waist circumference (WC), waist-to-hip ratio (WHR), calf or mid-arm circumference, and skinfold thickness, are easy to apply, inexpensive, and readily available. On the other hand, they show poor reproducibility and accuracy, and they do not necessarily reflect the distribution of body fat. BIA is a non-invasive approach that determines BC upon measurement of the electrical impedance offered by the different body compartments to the flow of an electric current. It is frequently used in clinical nutrition and allows to calculate fat mass, fat-free mass, and body cell mass (BCM) and to assess the hydration status. It is sensitive to shifts in fluid balance and is less reliable in settings like liver, kidney, and heart failure, or electrolyte disorders. DXA is emerging as the gold standard for the evaluation of BC (5), being widely available, relatively inexpensive, and highly reproducible. DXA requires the use of a low dose of ionizing radiation but allows to measure BC at the regional and whole body level. It uses a three-compartmental model that includes bone mineral content (BMC), fat mass (FM), and lean mass (LM). MRI and CT are the most accurate methods to study BC at the organ and tissue level. However, they are expensive, time-consuming, and often not easily accessible. CT also involves a non-negligible exposure to ionizing radiation. US is a valuable tool to estimate adiposity in clinical practice, since it is non-invasive, inexpensive, and portable, and it does not imply the use of ionizing radiation (6). Nevertheless, its accuracy is operator-dependent, and standardized sonographic procedures and indices are required to improve its reproducibility. US can be used to study BC at the organ and tissue level, including measurement of visceral and subcutaneous fat thickness at defined locations, but also at the cellular level, since intracellular fat (for instance, in hepatocytes or skeletal muscle cells) is associated with increased echogenicity. It appears clear that each of the methods that can be used for BCA has both advantages and limitations, so that the most appropriate technique should be selected based on the specific context.

BCA is now becoming increasingly popular both in the research field and in clinical practice. It is particularly relevant in the field of metabolism and clinical nutrition, but its sphere of application is potentially much broader, touching virtually every specialty in medicine. BCA can be applied to the study of physiological and paraphysiological conditions—such as aging (7), growth, or adaptations to physical activity in athletes (8)—and of many different diseases including obesity, diabetes mellitus, cancer, malnutrition, and sarcopenia, providing insights into their pathophysiology. In addition, BCA can be used to assess the effects of specific interventions, such as physical exercise or nutritional therapy (9, 10). Within this Research Topic, Chao et al. explored which factors are associated with muscle health deterioration in older adults, who were followed for 6 years to assess transition from robust status to dynapenia (low muscle function with normal muscle mass), presarcopenia (low muscle mass with normal muscle function), or sarcopenia (low muscle mass and function). Older age (HR: 1.08, *p* < 0.001) and body composition parameters, especially higher fat-to-muscle ratio (FMR) determined by BIA (HR: 1.73, *p* = 0.029), were positively correlated with transition to dynapenia. By contrast, serum albumin levels were negatively correlated with transition to dynapenia (HR: 0.30, *p* = 0.004). In addition, clustering of two or more of these three factors was associated with an increased risk of transition to dynapenia, with a sort of dose-response effect. In summary, this study highlighted that older age, obesity (assessed using surrogate body composition parameters), and malnutrition (assessed using serum albumin) were the main risk factors for muscle health deterioration in healthy elderly individuals.

Jung et al. performed a cross-sectional study using BIA on 356 community-dwelling elderly individuals, who were subdivided into four categories (control, dynapenia, presarcopenia, and sarcopenia) to analyze existing differences in muscle and fat mass at the arm and leg. The study showed significant variations in body composition according to sex in the dynapenia group (reduced muscle mass at the arm and leg in women; increased fat mass at the leg in men), supporting the possibility of using different approaches to prevent this condition in males and females.

Sun J. et al. relied on BCA by whole body DXA to investigate the correlation of prediabetes and type 2 diabetes mellitus (T2DM) with adiposity in 28,429 adult patients. Their cross-sectional study found that, after adjustment for potential confounders, individuals with prediabetes or T2DM had significantly higher total percent fat (TPF), trunk fat mass, android and gynoid fat mass, and android to gynoid ratio as compared with non-diabetic individuals. In patients with T2DM, increased disease duration was associated with decreased adiposity, possibly due to therapeutic interventions. Interestingly, in patients without diabetes or with prediabetes, all body composition outcomes were directly related to serum glucose levels and glycated hemoglobin (HbA1c) levels, while significant inverse associations were found in patients with T2DM between serum glucose or HbA1c and TPF. These findings suggest that the relationship between laboratory parameters and adiposity in T2DM is complex, so that good glycemic control does not necessarily translate into improved body composition parameters.

Kerkadi et al. examined the association between bone mineral density (BMD) and body composition determined using DXA in 2,000 Qatari women, mostly obese. The study found that total lean mass was positively correlated with BMD at the spine and femur, as well as with whole body BMD and T-score. By contrast, a weak negative correlation was observed between total fat mass and femur BMD or whole body T-score. After adjusted non-linear regression, the association between parameters of fat distribution and whole body T-score was shown to be non-linear, suggesting that despite increased mechanical loading on bones, increased adiposity may not be protective against osteoporosis, but rather contribute to a decline in BMD.

Of note, the possibility of using imaging techniques for BCA sets the ground for the so-called opportunistic evaluation of body composition, which relies on the exploitation of data from scans performed for unrelated clinical reasons, and may be considerably facilitated by automatic methods (11). In this Research Topic, Van Erck et al. evaluated the use of a fully automatic method to measure psoas muscle area at the L3 level in axial CT images from patients undergoing transcatheter aortic valve replacement (TAVR), showing good agreement with the reference manual method.

A key concept in BCA is that differences in body composition may have substantial prognostic meaning (12, 13) and considerable impact on the management of patients in clinical practice. This is exemplified by several articles included in the present Research Topic. Kim et al. examined the association between volume status (in terms of edema index determined by BIA) and body composition parameters obtained by DXA/mid-thigh CT or physical performance in patients undergoing hemodialysis. Patients with high volume status had significantly decreased muscle mass (in terms of thigh muscle area index) and physical performance compared with those with low or intermediate volume status. These associations were not dependent on nutritional or inflammatory status (assessed with serum albumin and C-reactive protein levels, respectively). In addition, a high edema index was correlated with increased mortality, which might be influenced by changes in body composition and physical performance. Sun L. et al. demonstrated that overhydration is associated with an increased risk of left ventricular hypertrophy (LVH) in patients with chronic kidney disease (CKD) stage 1–4 (*n* = 302). The respective odds ratios for LVH were 3.082 (*p* = 0.023) and 4.481 (*p* = 0.015) in the middle and highest tertiles of overhydration, compared with the lowest tertile. The association was even stronger in patients with CKD stage 1–2. In addition, Xie et al. investigated the relationship between body composition parameters and hyperuricemia in 271 obese children and adolescents. Percentage of skeletal muscle (PSM) and skeletal muscle mass (SMM) determined by BIA had the strongest association with the risk of hyperuricemia (OR = 1.221 and 1.179, respectively). Hip circumference, waist circumference, and body fat mass (BFM) were also positively correlated with hyperuricemia in the whole sample of patients. However, after adjustment for age and BMI, the association between BFM and hyperuricemia was no longer detected in both boys and girls. SMM thus appears to be a better predictor of hyperuricemia compared with BFM. In the study by Xiong et al. BIA was explored as a predictor of clinical outcomes in children admitted to pediatric intensive care unit (*n* = 231). The phase angle (PhA) by BIA was found to be an independent predictor of 90-day mortality (cutoff: 3.0°), being significantly higher in survivors compared with non-survivors. There was also a weak negative correlation between PhA and duration of mechanical ventilation. Yue et al. also conducted research on critically ill children admitted to pediatric ICU (*n* > 10,000), uncovering a U-shaped association between serum magnesium on admission and 28-day in-hospital all-cause mortality. The lowest risk of mortality corresponded to serum magnesium levels ranging from 0.74 to 0.93 mmol/L, with levels above or below this range increasing the risk of mortality. Xu M. et al. showed that the psoas muscle index (PMI = psoas muscle area at L3 cross-section measured with CT divided by height squared) is able to predict long-term (1-year) mortality in young male patients with acute-on-chronic liver failure (ACLF), being a protective factor (HR = 0.851) at univariate COX regression analysis. In patients aged ≤ 40 years, PMI could predict 1-year mortality independently of MELD score.

Moreover, the distribution of different body components may have an impact on drug pharmacokinetics, and therefore on tolerance, toxicity, and effectiveness of pharmacological treatment. Increasing attention is now being paid to the effects of decreased fat-free mass on the pharmacokinetics of drugs (14). Given that the total amount of a drug that moves from blood into its distribution compartment (mainly fat mass for lipophilic drugs and fat-free mass for hydrophilic drugs) depends on the size of this compartment, drug distribution will be affected by body composition. When a drug is administered to a patient with its relative distribution compartment smaller than normal, for instance a sarcopenic patient, the peak plasma concentration will be higher and the time for clearance lower than normal, leading to potentiated but shorter pharmacological effects (15). In these conditions, toxicity could be increased even in the setting of decreased clinical efficacy. Evidence in support of this concept is provided by the study from Ando et al. who investigated the prognostic significance of body composition parameters determined using CT in patients with Crohn's disease before the beginning of anti-TNF therapy. Their study demonstrated that clinical outcomes at 5 years from induction of anti-TNF therapy were significantly worse in patients with lower skeletal muscle index (SMI) or mesenteric fat index (MFI = ratio of visceral to subcutaneous fat area at L3 level) compared with patients with higher SMI or MFI, respectively. A second paper focusing on patients with Crohn's disease is the one by Li et al. who reported that the GLIM (Global Leadership Initiative on Malnutrition) criteria, which include body composition parameters, may be more appropriate to assess the nutritional status in patients with Crohn's disease, as compared with screening with the NRS-2002 (Nutrition Risk Screening) tool.

It must be kept in mind that body composition analysis is inherently complex, given the large interindividual and interethnic variability that exists in both physiological and pathological conditions. This explains why numerous standardized criteria and parameters have been proposed in literature for BCA. At present, some of these are still debated and subject to change. Xu Z. et al. compared the performance of two screening methods (SARC-F and SARC-CalF) for the detection of sarcopenia in adult patients (*n* = 689) with T2DM. They concluded that SARC-CalF (strength, assisting with walking, rising from a chair, climbing stairs, and calf circumference) had enhanced sensitivity and improved overall detection of sarcopenia as compared with SARC-F (SARC + falling). Ge et al. instead determined the optimal cutoffs for the diagnosis of sarcopenia in the older Chinese population, including those for low appendicular skeletal muscle index (ASMI) by BIA, low handgrip strength, and low gait speed. In addition, Scafoglieri et al. examined the relationship between VAT distribution ratios (VAT/SAT and VAT/SM) and anthropometric indices commonly used in clinical practice to evaluate BC, including WHR and WC, in a multi-ethnic population (*n* = 419). In both sexes, VAT distribution ratios were shown to have non-linear associations with age and with anthropometric measurements. These findings are relevant in that they suggest that the interpretation of changes in body composition cannot simply rely on linear extrapolations.

The present Research Topic collection also includes: an interesting review on the pathology and physiology of ileostomy (Ma et al.), which is associated with significant structural, functional, and microbiological changes at the intestinal level; a randomized controlled trial showing that individualized nutritional support in hospitalized patients with oropharyngeal dysphagia after stroke may improve swallowing function and maintenance of nutritional status during the first week of hospitalization (Yan et al.); and a systematic review and meta-analysis on the efficacy of glutamine supplementation in the treatment of severe acute pancreatitis (Dong et al.).

To conclude, as supported by the evidence included in this Research Topic, body composition analysis is emerging as a valuable instrument for the study and clinical evaluation of several different diseases, especially in the field of clinical nutrition and metabolism. Importantly, body composition parameters may have predictive and prognostic values as well as a strong impact on patient management.

## Author contributions

All authors listed have made a substantial, direct, and intellectual contribution to the work and approved it for publication.
